# Supplementary material for: Antibiotic Resistance and Biofilm Formation in Enterococcus spp. Isolated from Urinary Tract Infections
Source: Pathogens. 2022 Dec 25;12(1):34. doi: 10.3390/pathogens12010034 (PMC9863506; doi:10.3390/pathogens12010034)
Supplement: Supplementary file 1 [file pathogens-12-00034-s001.zip › pathogens-2085207-supplementary.pdf]

Supplementary Table S1. Antibiotic susceptibility disks (Oxoid, England) along with codes and potencies.

Antimicrobial classes codes	Antimicrobial classes	Antimicrobial agent	Abbreviation	Concentration/Disc
I	**β-Lactam**	Penicillin<br>Ampicillin	P<br>AMP	10unites<br>10 µg
II	**Glycopeptides**	Vancomycin<br>Teicoplanin	VA<br>TEC	30 µg<br>30 µg
III	**Aminoglycosides**	Gentamicin<br>Streptomycin	CN<br>S	10µg<br>300 µg
IV	**Oxazolidinones**	Linezolid	LZ	30 µg
V	**Tetracyclines**	Tetracycline<br>Tigecycline	TE<br>TGC	30 µg<br>15 µg
VI	**Nitrofurantions**	Nitrofurantoin	NI	300 µg
VII	**Fluoroquinolones**	Levofloxacin<br>Ciprofloxacin<br>Norfloxacin	LE<br>CIP<br>NOR	5 µg<br>5 µg<br>10 µg
VIII	**Macrolides**	Erythromycin	E	15 µg
IX	**Streptogramins**	Quinpristin-Dalfopristin	Q-D	15 µg
